# A review of European-funded projects implementing living labs for the sustainability of agri-food systems

**DOI:** 10.1038/s41597-026-07057-x

**Published:** 2026-04-16

**Authors:** Annapia Ferrara, Bastian Göldel, Sabrina Arcuri, Francesca Galli, Sonia Massari, Giulia Gallo, Chiara Mignani, Daniele Vergamini, Alessio Cavicchi

**Affiliations:** Department of Agriculture, Food and Environment. University of Pisa. Via Del Borghetto, 80 – 56124 Pisa, PI Italy

**Keywords:** Agriculture, Interdisciplinary studies

## Abstract

Increasing awareness of the complex challenges faced by our agri-food systems highlights the need to rethink current governance models. In this context, Living Labs (LLs) are gaining recognition as a key approach to participatory innovation, as they promote co-creation, experimentation, and the implementation of innovations in real-world settings, through the involvement of citizens, businesses, public authorities, and the scientific community. The growing uptake of LLs is also reflected in the support provided by major funding agencies, most notably the European Commission (EC), which has funded a wide range of LL-based initiatives through its programmes. Despite this momentum, it remains difficult to obtain a clear picture of the societal challenges that LLs have addressed so far. The lack of a centralized repository, together with the heterogeneous use of terminology associated with this approach, hinders the identification of existing initiatives. This database aims to fill this gap by identifying EC-funded projects in the context of agri-food systems that implement LLs, and by listing and classifying the terminology used to describe them. The overarching goal is to provide a useful resource for both practitioners and the scientific community, thereby fostering a better understanding of the social challenges addressed by LL projects and helping to pinpoint additional potential areas of application.

## Background & Summary

We can no longer ignore the need to seek new governance models to ensure the sustainability and resilience of our agri-food systems. Although much has been discussed regarding the contribution of agri-food systems to climate change, widespread environmental degradation, and social inequalities^1^, our longstanding adherence to a paradigm of economic growth has significantly hindered the adoption of solutions enabling humanity to thrive within theEearth’s finite resources^1,2^. Therefore, scientific evidence on planetary boundaries^3^ urges us to reassess our human needs within the broader ecological systems we inhabit^4^ and to reconsider our actions in light of inter-species, inter-generational, and intra-generational justice^5^. This, in turn, requires us to challenge current institutional norms and social models, in order to deliberately transform our economies into systems that are more resilient and environmentally sustainable, capable of promoting equal rights and opportunities, and of ensuring the essentials of life for all^4,6^.

Although such position is now widely recognized, significant challenges remain in translating it into practice. Indeed, the post-growth approach - which begins by acknowledging the current unsustainable situation in light of the planet’s limited resources^4,7^ - does not provide a concrete agenda for guiding transformative change^7,8^. Yet, it invites us to view productive systems as a means of supporting holistic well-being rather than as engines of pure economic growth^4,6^. For this reason, we need to reconsider our role within a system of complex socio-environmental interdependencies, and acknowledge that this complexity must lie at the heart of the transformation required^4,6^.

Researchers in the post-growth field have developed a range of theories informing this process^7,9^. Among the most influential is that advanced by Kate Raworth in the *Doughnut Economics*^4^, who defines that that reciprocity, participation and multi-actor collaboration can enable forms of change that foster a systemic understanding of the problem and help identify appropriate solutions^4,6^.

From this perspective, the complexity underlying the agri-food system transformation calls for the development of sustainable alternatives based on co-educational processes able to challenge the consolidated historical, political, and cultural norms^2,10^. Agroecology offers a concrete example of such a sustainable and transformative approach. It shows that producing high-quality food with reduced environmental impact depends, above all, on rethinking farms and agricultural practices within the broader socio-ecological system -the agroecosystem- in which they are embedded^11^: emerging from the integration of ecology and agronomic disciplines, agroecology has rapidly evolved into a set of social movements concerned with sustainable agriculture and rural development that place food sovereignty at the centre, preserve traditional agri-food practices, protect biodiversity and soils, and value local knowledge^11–13^. Accordingly, this transition process requires support not only for innovation in on-farm practices and processes, but also for the creation of new knowledge, and for new ways of generating and disseminating it^2,10,14^. For instance, questions such as “What is soil health and how can it be measured?” or “How can biodiversity be preserved?” call for a more horizontal and integrated approach involving different actors -farmers, scientists, policymakers and citizens- who, by bringing together different forms of knowledge, expertise and experiences, are able to co-develop new actionable knowledge in support of long-term sustainability^2,10,14^.

In this action-oriented transformative process, research itself acquires new value and assumes new roles^2,10,15^. For instance, by addressing complex challenges, the agroecological movement requires a strong transdisciplinary approach, namely, an observational and operational lens that transcends individual disciplines and integrates diverse knowledge systems^15,16^. In this sense, the broader range of scientific disciplines is brought together with experiential, indigenous, political, and other forms of knowledge, recognising that academic knowledge does not hold inherent epistemic superiority over other ways of knowing, but rather coexists with them as equally valid^15,16^. Indeed, within a post-growth understanding of our agri-food systems, it is essential that the educational process, including for researchers, be experiential and grounded in pedagogies that foster critical awareness of the policies and infrastructures underpinning agri-food transformation^2^. Accordingly, we are witnessing stronger involvement of the social sciences and humanities (SSH) alongside the natural sciences^15^, as well as an increasing use of participatory methodologies, such as participatory action-research, which go beyond analysing different viewpoints, and engage, instead, in processes of reflexivity and action within different knowledge systems^15,16^. What Fernández González and colleagues describe as “activist transdisciplinarity,” positions science in the service of communities, within a research process that fosters a collective empowerment, since it begins with issues posed by the local communities, and proceeds with co-constructed inquiry^16^.

In this context, *Living Laboratories* or *Living Labs* (LLs) have become recognized instruments for fostering change, and now play a significant role in discussions on sustainable transitions^17^. Defined as “user-centered, open innovation ecosystems”^18^, LLs facilitate the experimentation of solutions aimed at addressing critical contemporary challenges by engaging the actors of the public–private–people partnership^18,19^ in a collective learning and co-creation process that accelerates the transition toward more equitable systems^17^.

For this reason, LLs have been applied across a wide range of domains^20,21^, with their use expanding rapidly in the context of sustainable agri-food systems^17,22–25^. As with any new entity emerging in a given territory^26^, LLs have peculiar features and objectives, and act in specific ways^22–25^. For example, the so-called “Agroecology Living Labs” foster broad interrelations between urban and rural areas within city-region food systems, transcending geographical and administrative boundaries, and connecting the elements of the agri-food system that collectively shape such activities^22^. At the same time, the local context strongly influences the characteristics of the Labs: first and foremost, the development of farming technologies is influenced by the local climate and seasonal conditions^22^, local agroecological priorities, and specific territorial constraints^24^. Moreover, transformative processes are shaped by both territorial and actor-scale dimensions^23,24^. This leads to two important considerations. First, results must be communicated effectively to ensure that all knowledge systems involved can contribute to transformation, and that co-created outcomes become embedded in the sociocultural context^23^. The second consideration concerns the organizational and institutional structures^24,25^. Indeed, although grassroots and civil society organizations can provide alternative arrangements to rebalance power dynamics, decentralized and innovative coalition models -such as the Agricultural Knowledge and Innovation Systems (AKIS)- may enable large-scale transformation^25^, and create greater opportunities for less-heard voices to influence decision-making processes^24^.

As spaces driving post-growth approach and practices for our agri-food systems, it is no surprise that LLs have increasingly attracted international funding^6^. In particular, since the early 2000s, the European Commission (EC) has supported and invested in this approach, promoting, among other initiatives, the European Network of Living Labs (ENoLL), which has been established under the Council of the European Union. Recognised today as an independent no-profit association, ENoLL is one of the first international institutions aimed at fostering collaboration and disseminating knowledge about LLs. By convening actors around the ambition of building a “greener, healthier, more inclusive, and resilient continent”^27,28^, LLs are increasingly being mobilised as a key approach to supporting the sustainable transition of agri-food systems, having agroecology-oriented LLs as a prominent domain of application. For instance, the *Agroecology* *Partnership*, is a recent co-funded programme by the EC and 72 partners across 26 Member States, Associated, and Third countries. The programme funds high-level research conducted by agroecology LLs and research infrastructures, with the aim of accelerating the transition of farming systems towards greener and more sustainable models^24,25^.

After more than twenty years of investment in this approach, there are still many open questions, such as “How do LLs guide the sustainability transition process?” and “What results have actually been achieved?” To answer these questions, it is necessary not only to compare multiple LLs, but also to evaluate them at different scales^24^. However, in attempting to answer these questions, two major obstacles emerge:

1. First, to date, there is no centralized platform or repository that collects and organizes information on existing LLs. A repository could serve as a resource not only for the scientific community, but also formultiple stakeholders interested in this approach.
2. Second, there is a wide variety of terms used to refer to the “Living Lab” approach^26^. This complicates the task of identifying and retrieving data on LLs.

Considering these challenges, our research aims to address the primary need to identify the EC-funded projects in the agri-food domain that adopt the LL approach, with the dual objective of mapping the terminology used and the challenges addressed.

## Methods

To address this aim, we conducted a review of EC-funded projects that included the implementation of LLs in response to the challenges of agri-food systems. Indeed, project reviews, as well as project portfolio analysis, have proved to be particularly useful for capturing project achievements and identifying outstanding research and innovation needs^29^. To this end, we carried out a *Systematic Research Project Review*^30,31^. Adapted from the traditional systematic reviews of scientific literature, this methodology is designed to accommodate the heterogeneity and complexity of project-derived information, which is often dispersed across different structures, formats, and sources^30,32–34^.

The review process, including the analysis of results,was conducted between July 2023 and November 2024. It comprised several phases, interspersed with moments of discussion and reflection within the research team, which played a crucial role in strategically guiding the review and shaping the decisions made in later stages.

### Data collection

For our review, we drew inspiration on the steps proposed by García-Holgado and colleagues^30^. These steps include formulating a reliable query string prior to the review process, defining inclusion and exclusion criteria for the results, and creating a database for data collection.

For the review, we used the EC’s open-source CORDIS repository (accessible at https://cordis.europa.eu/search). CORDIS provides comprehensive metadata on EC-funded research and innovation projects, including funding programmes, calls, project reports, outputs, and deliverables. The use of this repository is well suited to the purposes of this study, as the editorial content published on CORDIS is provided under the Creative Commons Attribution 4.0 International License, which permits both sharing and adaptation for research purposes.

Following an initial stage of familiarization with the CORDIS repository, we conducted a targeted search to identify all funding calls that supported projects related to agri-food systems, their specific sectors and related activities. To do so, we used the keywords “agro*”, “agri*”, and “food*” interposed by the Boolean operator “OR”^35^.

Simultaneously, we applied a search filter to display only calls specifically related to the “Horizon 2020” and “Horizon Europe” programmes. The former (H2020) covered the 2014–2020 funding period, while the latter (HEU) covers the current 2021–2027 funding period. Indeed, compared to earlier “Framework Programmes” funded since 1984, the two most recent programmes provide a stronger nexus between research and innovation in order to address global challenges and foster a sustainable transition, which makes H2020 and HEU more aligned with the objectives of our study^36^.

As of November 2023, we identified 1,117 calls funding projects in the context of agri-food systems. After excluding 38 calls reported in languages other than English, individual projects were extracted from each funding call.

Thereafter, we carefully read and analysed each project’s abstract to identify projects that implemented LLs strictly within the context of agri-food systems, excluding those focused on other broad domains, such as rural development. To identify these projects, we adopted a qualitative approach rooted in content analysis^37,38^. This analysis was deductive and informed by the six interrelated dimensions that characterize LLs, as defined by the ENoLL^18^. The dimensions are as follows:

- *Active user involvement*. In LLs, end users are not passive recipients of innovations. Rather, they actively participate in the conception, testing, and validation of solutions. This approach helps ensure that the results are genuinely useful and relevant to those who will use them. Active user involvement also aims to foster a stronger sense of ownership of the co-creation process and a shared responsibility among participants, thereby increasing not only the likelihood of long-term success of the innovation but also the overall participants’ empowerment from the process.
- *Multi-stakeholder participation*. An intrinsic component of the LL process is the involvement of all actors of the so-called Quadruple Helix, namely civil society, businesses, governments, and research institutions, or the Quintuple Helix, which, according to some scholars, also includes the environment^39^. For this reason, LLs require well-defined mechanisms to ensure effective interaction among actors and their different perspectives, skills, and knowledge that will guide the creation of more sustainable solutions. Therefore, it is crucial for LLs to structure collaborative processes that enable continuous dialogue among all stakeholders.
- *Co-creation*. In LLs, co-creation is based on the principle that stakeholders actively participate throughout the entire process, from collective inquiry to the testing or implementation of the solution. In this context, all actors are considered necessary and equal contributors. This dimension highlights that the combination of different knowledge and perspectives is resulting in a new knowledge co-created from an inclusive and collaborative innovation process.
- *Multi-method approach*. There is no single methodology applicable to LLs. Each LL must combine and adapt user-centric methodologies that best suit its objectives. This flexible approach allows for the use of various tools and methods, such as design thinking, action-research, digital co-design tools, and other participatory techniques, but also different theories and disciplines in order to address each specific challenge.
- *Real-life setting*. Activities in LLs must take place in real-world settings to ensure a thorough understanding of the dynamics within their operating environments. Operating in real-life settings enables the collection of reliable data, and allows for assessing the impacts of innovations in the contexts where they are produced.
- *Orchestration*. An LL is not just a meeting space for social actors. Orchestration refers to the internal mechanisms that enable the LL to serve as a springboard for interactions with external actors as well, positioning it as a key node within a broader innovation ecosystem. Therefore, orchestration ensures that the LL is not an isolated entity, but rather a catalyst for innovation processes that also engage actors outside its immediate boundaries.

To proceed with the qualitative text analysis, two of the authors independently read the individual project abstracts. During this process, each author identified the presence or absence of elements in the abstracts that could be attributed to one of the dimensions characterizing an LL, as defined by the ENoLL^18^. The presence of elements corresponding to each dimension was marked with an “X” in a dedicated analysis grid, whereas the absence of explicit evidence was left blank. At the same time, the portion of text referring to the individual categories identified in the abstract was extracted and linked to the corresponding ENoLL dimension.

Indeed, applying the six ENoLL^18^ dimensions used to define an LL was crucial for identifying the projects relevant to this study. First, it enabled us to verify the actual presence of an LL, moving beyond the mere occurrence of the term “Living Lab” or related expressions in the project abstracts, and thereby reducing the risk of self-referentiality. Second, it allowed us to recognise projects that did not explicitly mentioned the implementation of an LL but nonetheless displayed the core characteristics of the approach. However, we must highlight that during the review process, we found that not all project descriptions displayed all six ENoLL^18^ dimensions. Overall, this phase resulted in 281 projects potentially eligible for inclusion in our study.

It is important to note that CORDIS project descriptions typically provide a concise yet substantive information on project objectives, approaches, methods, and expected results, which proved sufficient for identifying references to each LL dimension. Nonetheless, the depth and completeness of the available information are constrained not only by the strict word limits inherent to the abstract format, but also by the intrinsically prospective nature of project abstracts. Indeed, these texts primarily articulate applicants’ intended activities and anticipated achievements, rather than providing systematic evidence of what is ultimately implemented during project execution.

For this reason, we cross-validated the results using project websites or the corresponding deliverables available in the CORDIS repository. Reviewing secondary sources was crucial for corroborating the actual implementation of the LL approach within each project. Evidence of LL adoption emerged most clearly from explicit descriptions of transdisciplinary dialogue mechanisms and from well-defined arrangements for stakeholder engagement, governance, and the management of co-creation processes. These elements are central to an LL, as they operationalise its open innovation logic and distinguish the LL from more generic multi-actor approaches^18^. Consistent with the EC’s guidance on the use of LLs in Horizon projects, and in line with the PREMIERE project’s indications on “preparing multi-actor projects in a co-creative way”, such characteristics are expected to ensure the robustness and continuity of the activities within an LL, compared to more generic project-bound multi-actor networks^40,41^.

Consistent with the aim of this study, we subsequently excluded all projects whose CORDIS page contained no explicit focus on agri-food systems, relevant sectors, or related business activities either in the project abstract, title, or in the project keywords. This step was necessary because, in a number of cases, terms such as “*food”* or “*agr-”* appeared only because of the specific funding programme under which the project was funded, rather than reflecting projects' substantive focus on agri-food systems. As will be discussed in the following sections (see Data Overview for further details), funding priorities addressing agri-food often include adjacent domains, such as the bioeconomy or, more broadly, natural resources, thereby not necessarily implying that the project focuses directly on the agri-food systems. Overall, the application of these two exclusion criteria resulted in the exclusion of 92 projects from the initial 281 selected in the first step.

Finally, we conducted two verification steps to ensure that all projects relevant to the objectives of this study had been selected. First, we extracted from the abstracts of the selected projects the keywords associated with the LL terminology (see Table 3 for details) and used them to construct a dedicated search string for use within the CORDIS repository. We then applied the same screening procedure and the same inclusion and exclusion criteria described above to the resulting set of records.

**Fig. 1:**
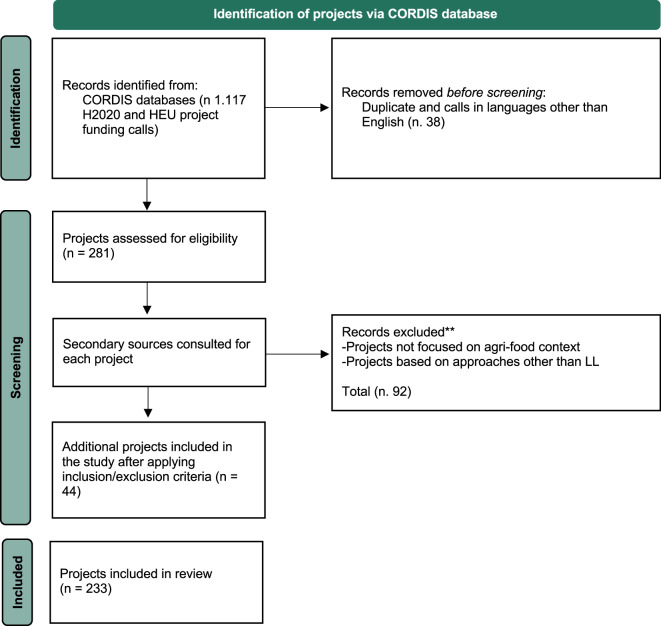
Visual representation of the sequential steps adopted for the collection and verification of results in our systematic review of projects. Authors' elaboration based on the Preferred Reporting Items for Systematic Reviews and Meta-Analysis (PRISMA) workflow^42^.

Second, we performed a final cross-check to minimise the risk of oversights due to the handling of a substantial volume of information, as well as potential technical issues affecting the display of results when running specific search strings. To this end, we consulted the secondary sources associated with each project included in the final sample and recorded any related H2020 and HEU projects, often mentioned as “sister” or “twin” projects. These projects were subsequently screened using the same steps and inclusion and exclusion criteria. After these verification steps, 44 additional projects were identified. At the end of the review process, we included a total of 233 projects in our study. Figure 1 provides a schematic overview of the sequential steps used to collect and validate the results emerging from our systematic project review.

All selected projects were recorded in a dedicated Excel file. For each project, we examined the related CORDIS page to extract specific information, including the related LL terminology used, and the societal issue addressed. It is important to remind that our research does not examine the individual LL experiences developed within each project. Rather it adopts a higher-level perspective by identifying projects that implement the LL approach, and analysing their main characteristics.

## Data Records

Our database^43^ is available in its current second version at the following link: 10.5281/zenodo.18538508^43^. The database is provided as an Excel file comprising two sheets, “Project characteristics” and “Project Overview”, which contain data gathered from the 233 selected projects. The extracted data are organized into specific columns. In the cells corresponding to each column heading, a legend is also provided to help users navigate the dataset. Below, we present an overview of the two sheets, together with a preliminary analysis of their content.

### Project characteristics

Within the “*Project characteristics*” sheet, the first two columns refer to the acronym (“*Project Acronym”*) and the title (“*Project Title”*) of each selected project, while the third column (“*URL”*) contains the corresponding web address. We decided to include both project titles and acronyms to ensure project identification. Indeed, acronyms are often the primary identifiers of EC-funded projects, whereas the full titles provide essential context for understanding their focus. The CORDIS repository provides both elements, thereby facilitating searches for users who might be familiar with only one of the two.

The following columns, from the fourth to the twenty-second, provide information on the inclusion criteria applied to select the projects.

In particular, because the selection focused on projects related to agri-food systems, the fourth column *(“Agri-food focus”*) uses “YES” to identify the projects that focus exclusively on the domain of agri-food systems and “RELATED” for those in which agri-food represents one of several areas of investigation for the project, alongside health, transport, or manufacturing, for example.

Columns five to twenty-two indicate the presence of elements related to the six-dimensional ENoLL framework^18^ within the selected project abstracts. More specifically, the database includes columns capturing three levels of information in this regard. The first set of columns (“*Active user engagement”, “Multi-method approach”, “Multi-stakeholder participation”, “Orchestration”, “Real-life setting”, “Co-creation”*) indicates, through an “X”, the presence of textual references in the project abstract related to a specific LL dimension. The subsequent set of columns (“*Extracted text from the project abstract”*) reports the keywords or excerpts associated with that LL dimension, as extracted from the project abstracts. The third set of columns (“*Emerging themes”*) reports a thematic classification of the information emerging from the text across the different dimensions for each project. To this end, we carried out a thematic analysis^37,38^ of the abstracts to identify recurring themes related to the different LL dimensions. To do so, we first grouped relevant information under each dimension. Following an initial familiarisation with the textual content, we identified themes by applying a qualitative coding analysis^37,38^. Below, we provide an overview of the themes emerging for each dimension:

- *Active user involvement*: the information related to this dimension revealed three main themes. The first concerns the approach to active user engagement. This theme includes information on the co-creation process, from stakeholder engagement and knowledge exchange, to the testing and validation of the solutions developed (labelled in the database as “A”). The second concerns the stakeholders at the centre of the co-creation process (labelled in the database as "B”). In particular, generic mention to stakeholders, such as “relevant stakeholder(s)”, “multi-stakeholder”, or the “helix”, are labelled in the database as “B generic”, whereas specific categories of actors, such as farmers, farm advisers, or policymakers, are labelled as “B specific”. The third theme captures cases in which comprehensive information is provided both on the approach to active user engagement and on the stakeholders involved. This theme is coded in the database as “C specific” when the stakeholders are explicitly identified, and as “C generic” when their category is not specifically identified.
- *Multi-stakeholder participation*: within this dimension, two themes emerged. The first refers to the presence of relevant stakeholders whose interaction contributes to the exchange of different knowledge systems (in the database labelled as “Theme 1”). The second refers to the broader concept of the “multi-actor” approach, or to the “triple”, “quadruple”, and “quintuple helix”, depending on the context of the issue being addressed (labelled as “Theme 2”). It is within this dimension that the nature of the different LLs becomes more evident. Indeed, this dimension encompasses different experiences, from pilot cases in agriculture that are more corporate-oriented, to cases that place greater emphasis on citizen involvement to address broader food system challenges.
- *Co-creation*: within this dimension, three themes emerged. The first concerns an explicit reference to the co-creation process, including information on co-design and, more generally, on collaboration in the development of new solutions (labelled in the database as “Theme 1”). The second relates to tools and approaches that enable the co-creation process (labelled in the database as “Theme 2”). The third encompasses cases in which the co-creation process is less evident and the focus is instead placed on activities that support the development or transfer of solutions, such as data collection or the implementation of a process or technology (labelled in the database as “Theme 3”).
- *Multi-method approach*: within this dimension, two main themes emerged. The first (D1) concerns the participatory, transformative, and action-oriented approach. More specifically, this theme is divided into two sub-categories, which capture, respectively, the inter-, trans-, or multi-disciplinary approach shaping the dialogue between different knowledge domains (labelled in the database as “D1-TRA”), and the theories underpinning transformative, participatory, and action-oriented approaches (labelled in the database as “D1-TH”). The second theme (D2) concerns the set of information related to tools, techniques, and process activities that support co-creation. This theme is divided into two sub-categories: the first refers to specific tools and methods, including, for example, scenario and foresight techniques (labelled in the database as “D2-TOOL”). The second refers to activities that enable the co-creation process, such as workshops, events, or peer-to-peer learning (labelled in the database as “D2-PROC”).
- *Real-life setting*: within this dimension, four themes emerged as representative of the information collected from the project abstracts. The first concerns the territorial context in which the process of co-creation, testing, or replication of the solution is anchored. This theme is represented by information on specific territories, but also includes references to “relevant regions”, “areas with high potential”, or regions with specific agricultural productions (labelled in the database as “A”). The second theme concerns the value chain or process context in which the co-creation process or solution implementation is anchored. In this case, a specific part of the operational setting is explicitly specified, such as an entire value chain or part of it (labelled in the database as “B”). The third theme relates to information on virtual co-creation and experimentation environments (labelled in the database as “C”). The fourth and final theme concerns general settings in which innovation and experimentation take place (labelled in the database as “D”). This includes information on case studies, demonstrations, and meta-experiments, as well as stakeholders interactions in platforms for which the real-world context, the operational setting, and the virtual experimentation environment are not specified.
- *Orchestration*: This dimension is structured around two overarching thematic areas, relating to communication and governance mechanisms within and beyond the LL. Each area is further articulated into two sub-categories. First, internal orchestration (T1) comprises, on the one hand, information concerning the structure of internal collaboration, not only in terms of establishing and organising partner networks, but also with regard to governance mechanisms, coordination roles, and backbone organisations (labelled in the database as “T1.1”). On the other hand, it includes information on collaborative processes, understood as the mechanisms and procedures through which actors work together in the LL context, encompassing, for example, communities of practice, knowledge communities, and capacity-building activities (labelled in the database ad “T1.2”). Second, external orchestration (T2) covers information on external linkages, innovation diffusion and scaling dynamics, such as connections with cities, regions, other countries, or wider networks, hubs acting as “injection points”, scaling trajectories and knowledge transfer processes, interregional “belts”, and platforms enabling service provision at the national or European level (labelled in the database as “T2.1”). Second, it includes information on outward-facing communication and dissemination strategies, including visibility campaigns as well as tools and channels used to disseminate results and the innovation produced (labelled in the database as “T2.2”).

### Project overview

The second sheet contains project metadata, including the project start year, country, coordinating institution, and allocated budget, together with selected text elements extracted from project abstracts. Notably, the third column (titled “*Horizon Programme*”) identifies the funding programme (H2020 or HEU) under which the selected projects were supported.

The fourth column (“*Starting point”*) indicates the starting year of each project. It is important to specify that, in our database, this refers to the year in which project activities formally started, rather than the year in which the grant agreement was signed by the EC.

The fifth column (“*Main Programme”*) refers to the different “pillars” representing the areas of project funding. As shown in Table 1, the structure of the H2020 and HEU programmes is organised into specific pillars, each corresponding to a broad area of intervention and to the related priority clusters. In general, there are similarities between the structures of the two programmes, as the HEU programme was developed on the basis of its predecessor. However, significant differences also exist, particularly with regard to pillars II and III. In H2020, these are identified as “Industrial Leadership” (pillar II) and “Societal Challenges” (pillar III). In contrast, in HEU they are combined into a single pillar called “Global Challenges and European Industrial Competitiveness”. This title emphasises the close relationship between economic sectors and society as a whole, as well as the complexity and the global significance of the challenges addressed.

**Table 1:** Structure of H2020 and HEU project funding areas. Authors’ elaboration.

H2020		HEU	
*Pillar*	*Cluster*	*Pillar*	*Cluster*
1. Excellent Science	1.1. European Research Council	1. Excellent Science	1.1. European Research Council (ERC)
	1.2. Future and Emerging Technologies		1.2. Marie Skłodowska-Curie Actions (MSCA)
	1.3. Marie Sklodowska-Curie Actions		1.3. Research Infrastructures
	1.4. Research Infrastructure (including c-Infrastructure)		
2. Industrial Leadership	2.1. Leadership In Enabling and Industrial Technologies	2. Global Challenges and European Industrial Competitiveness	2.1. Health
	2.2. Access To Risk Finance		2.2. Culture, Creativity, and Inclusive Society
	2.3. Innovation in Smes		2.3. Civil Security for Society
3. Societal Challenges	3.1. Health, Demographic Change, and Well-Being		2.4. Digital, Industry, and Space
	3.2. Food Security, Sustainable Agriculture and Forestry, Marine and Maritime and Inland Water Research, and The Bioeconomy		2.5. Climate, Energy, and Mobility
	3.3. Secure, Clean, and Efficient Energy		2.6. Food, Bioeconomy, Natural Resources, Agriculture, and Environment
	3.4. Smart, Green, and Integrated Transport	3. Innovative Europe	3.1. European Innovation Council (EIC)
	3.5. Climate Action, Environment, Resource Efficiency and Raw Materials		3.2. European Innovation Ecosystems
	3.6. Inclusive, Innovative, and Reflective Societies		3.3. European Institute of Innovation and Technology (EIT)
	3.7. Secure Societies - Protecting Freedom and Security of Europe and Its Citizens	4. Widening Participation and Strengthening the European Research Area	4.1. Widening Participation and Spreading Excellence
4. Spreading Excellence & Widening Participation			4.2. Reforming and Enhancing the European R&I System
		5. Euratom	5.1. Fusion
5. Science with and for Society			5.2. Fission
			5.3. Joint Research Center

The sixth column (“*Related call for proposal”*) reports the call for proposals associated with each project. In this context, calls for proposals refer to the funding opportunities issued by the European Union (EU) bodies in the form of grants awarded to third parties, whose activities and outputs contribute to the EU policy objectives.

The seventh column (“*Funding Scheme*”) reports the funding scheme under which each project was funded. The database includes projects funded under Research and Innovation Actions (RIA), which primarily support the generation of new knowledge and the advancement of exploratory research. Accordingly, RIAs typically aim to develop novel solutions and technologies and may include experimental validation of early-stage concepts^44,45^. We also identified Innovation Action (IA) projects, which are oriented towards activities closer to market and therefore place greater emphasis on the development and validation of innovations. They are based on more mature concepts compared to RIAs, and aim to the construction and testing of prototypes, demonstrators, or pilot implementations in operationally relevant environments^44,45^. Within this category, one project was funded under the JU-IA scheme, that is, an Innovation Action implemented through a Joint Undertaking (JU). JUs were established under H2020 as large-scale public–private partnerships. Similarly,under HEU, they continue as institutionalised European Partnerships, with the aim of addressing major societal challenges while strengthening Europe’s industrial competitiveness through coordinated research and innovation activities including prototyping and large-scale demonstrations^44,46^. In addition, the implementation of LLs in agri-food contexts was substantially supported through Coordination and Support Actions (CSA). CSAs are primarily geared towards networking, coordination, and capacity-building, rather than direct research or technology development. They typically facilitate knowledge exchange and ecosystem mobilisation through activities such as workshops and conferences, stakeholder coordination platforms, training and education initiatives, and the communication and dissemination of project results. Finally, one project was funded under the Marie Skłodowska-Curie Actions (MSCA), the EU’s flagship programme for researchers’ career development, which supports structured training pathways and international and intersectoral mobility. The MSCA is strongly people-centred and aims to enhance researchers’ competences through exposure to diverse research and innovation environments and through knowledge transfer across disciplines, sectors, and countries^44–46^

The eighth column (“*EU Contribution”*) reports the amount offunding allocated to each project. It is important to note that, in our database, this column refers exclusively to the financial contribution provided by the EC, and therefore does not account for any additional resources or co-funding secured from external sources.

Columns nine (“*Coordinator”*) and ten (“*Coordinating country”*) report, respectively, the coordinating organisation for each project and the country of the coordinating institution.

Subsequently, columns eleven and twelve (grouped under “*Living Lab terminology*”) capture the terminology used to describe the LL approach across the projects included in our database. These terms were extracted from the project abstracts available in CORDIS. Specifically, column eleven records the wording directly derived from the expression “living lab” (including grammatical variants and compound formulations), whereas column twelve compiles all other terms mentioned in each project abstract that refer to similar concepts.

Finally, column thirteen (“*Societal Issue”*) reports the societal need around which each project was built. The cells in this column contain information manually extracted from the abstracts of the projects selected from the CORDIS repository. The text has not been altered or analszed..

## Data Overview

In this section, we provide a preliminary analysis of selected data extracted from the projects included in our database.

First, it should be noted that the database mostly includesprojects whose primary focus is related to the agri-food systems (71.12%), as compared with projects that address the agri-food systems as an empirical application area alongside other thematic domains (28.88%). Among the projects, two also involve the participation of the ENoLL, namely, one H2020-funded project (ALL-READY) and one HEU-funded project (FARCLIMATE).

**Fig. 2:**
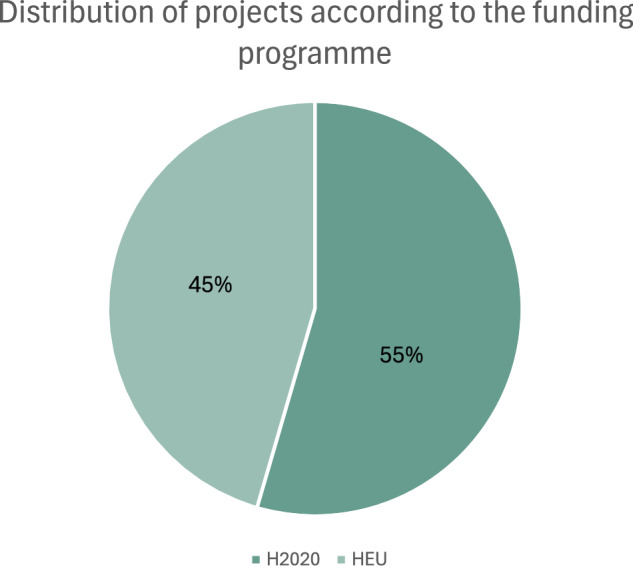
Visual representation of H2020 and HEU projects within the agri-food system context implementing the LL approach. Authors’ elaboration based on the results ranging from 2014 to 2024, derived from our systematic project review.

With respect to the funding programme supporting each project, the distribution shows that just over half of the projects (exactly 54.5%, corresponding to 127 projects) fall under H2020, while the remaining 45.5% (corresponding to 106 projects) fall under HEU. Figure 2 below provides a visual overview of the distribution of projects by funding programme.

**Fig. 3:**
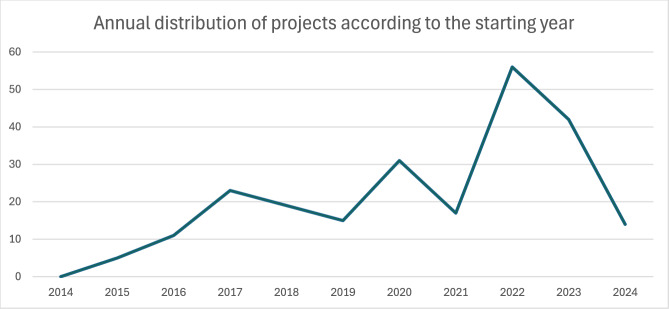
Annual distribution of agri-food-related projects implementing the LL approach within H2020 and HEU programmes. Authors’ elaboration based on results from the systematic project review.

This distribution clearly reflects the growing prominence of the LL approach during the most recent programming period. More specifically, an examination of the column related to projects start year reveals a relatively stable trend in the funding of projects implementing LLs, although a notable decline is evident in 2021, likely due to the introduction of the new HEU programme (2021–2027), before peaking in 2022. Evidently, part of the reason is to be attributed to specific strategic priorities defined for the HEU programme, which have had substantial influence on the implementation of LLs within projects. This is particularly evident for the *Mission Soil* strategy and its related calls for proposals aiming to reach the establishment of 100 LLs across Europe in support of the ecological transition. Figure 3 provides an overview of the percentage of projects starting in each year from 2014 to 2024.

As explained in the methodological section, it is important to note that data collection for this study was completed at the end of 2023, which may account for the apparent decline of funded projects that started in 2024.

With regard to the funding pillars (EU's priorities for research and innovation), 2.15% of the H2020 projects were funded in the area of technologies with industrial applications, specifically nanotechnologies (H2020-EU-2.1.1.). Most of the remaining projects fall under the Societal Challenges pillar, primarily within cluster 3.2., which addresses issues such as “Food security, Sustainable Agriculture, Forestry, Marine and Inland Water Research, and the Bioeconomy”. The aim of this cluster is to promote sustainable and resilient agri-food systems while ensuring food security and the responsible management of natural resources. To a lesser extent, projects were also funded under cluster 3.5., which targets "Climate action, Environment, Resource Efficiency, and Raw materials”, with the aim of enhancing environmental sustainability and addressing climate change through efficient resource management. Similarly, within HEU, the majority of projects received funding under cluster 2.6. (Food, Bioeconomy, Natural Resources, Agriculture, and Environment). Together with cluster 3.2. from the previous programme period, these areas account for 84.12% of all projects implementing LLs. By contrast, 3.1% of projects were funded in the energy sector. Specifically, H2020 cluster 3.3. (0.86%) relates to “Safe, Clean, and Efficient Energy” and aims to support the transition to renewable sources and low-impact solutions. In addition, HEU cluster 2.5. (2.15%) focuses on “Climate, Energy, and Mobility”, with the aim of developing innovative and sustainable energy systems to address climate change. For a visual overview of the clusters under which the selected projects were funded, see Table 2.

**Table 2:** Number and related percentage of agri-food-related projects funded under specific EU's priorities for research and innovation withinH2020 and HEU programmes.Authors' elaboration based on results from the systematic project review.

H2020	n.	%	HEU	n.	%
H2020-EU.1.3.	1	0.43			
			HORIZON.1.3.	1	0.43
H2020-EU.2.1.1.	5	2.15			
H2020-EU.2.1.2.	1	0.43			
H2020-EU.2.1.6.	1	0.43			
H2020-EU.2.3.	3	1.29			
H2020-EU.3.2.	95	40.77	HORIZON.2.6.	101	43.35
H2020-EU.3.3.	2	0.86	HORIZON.2.5.	5	2.15
H2020-EU.3.5.	14	6.01			
H2020-EU.4.b.	1	0.43	HORIZON 4.1.	2	0.86
H2020-EU.5.d.	1	0.43			

Authors’ elaboration on results. In this table, results are arranged following a structure that encourages to visualise similarities in the themes between the H2020 and HEU programs.

**Fig. 4:**
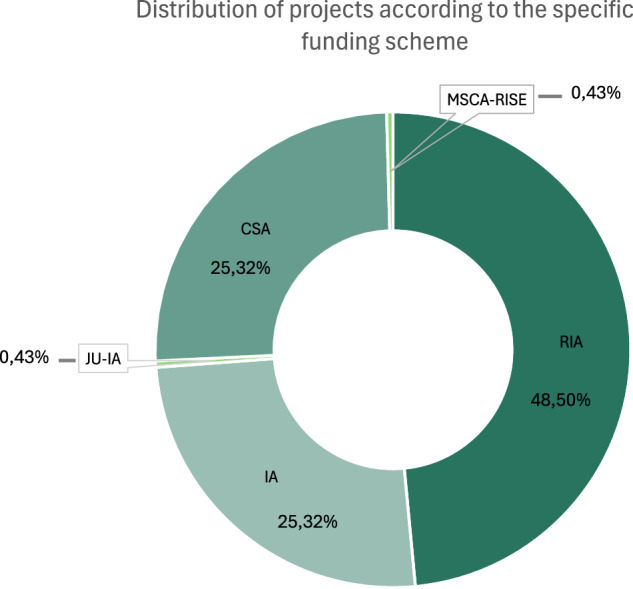
Distribution of agri-food-related funded projects implementing the LL approach according to the specific funding schemes for H2020 and HEU programmes. Authors’ elaboration based on results from 2014 to 2024 derived from the systematic project review.

Understanding the funding schemes associated with each project provides insights into the nature of the project itself, its strategic objectives, and the maturity level of the solution or technology developed, commonly referred to as the Technology Readiness Level (TRL). TRL is a classification system ranging from TRL1, at which basic principles are observed, to TRL9, at which the system (the innovation proposed) has been tested and is ready for the market^43^. Figure 4 provides an overview of the percentage distribution of funding schemes across the different projects.

The distribution of funding schemes across the database provides useful indications of the maturity level and the dominant functions attributed to LLs in agri-food systems. The prevalence of projects funded as RIAs (48.50%) suggests that LLs are most frequently embedded in initiatives primarily oriented towards exploratory research and knowledge generation. Indeed, the expected outcome of an RIA is often a functional prototype or validated technology up to TRL6 (corresponding to a technology demonstrated in a relevant environment). In this respect, LLs appear to be used predominantly as environments for concept development and early validation, in which novel solutions can be tested under controlled or semi-controlled conditions without the expectation of immediate market deployment^43^. At the same time, the share of IAs (25.32%) indicates that, albeit less frequently, LLs are also mobilised in trajectories closer to market, where the aim is to consolidate more mature concepts into prototypes, demonstrators, or pilot implementations in operationally relevant settings^43^. Indeed, IAs projects seek to achieve higher TRLs than RIAs projects do. The presence of a JU-IA (0.43%) further reinforces this interpretation by indicating that LL-based approaches can also be integrated into large-scale public-private partnership initiatives characterized by stronger industrial governance and higher expectations in terms of technology maturity. Indeed, JUs are intended to address major societal challenges within Europe’s industries by combining EU funds with contributions from industry and other partners, thereby aligning with sectoral roadmaps and supporting TRL progression to higher levels^46^.

In addition, CSAs, which are not structured around a predefined TRL level, account for a share comparable to that of IAs projects (25.32%), highlighting that LL implementation is associated not only with technology development, but also with coordination, networking, and capacity-building mechanisms across countries^44,45^. Finally, the marginal presence of MSCA- RISE projects (0.43%) indicates that researcher-centred training and mobility pathways are not a dominant channel for LL-related work in this dataset. Nonetheless, the MSCA project funded in 2021 still provides evidence of the interest in supporting activities that help strengthen researchers' interdisciplinary and knowledge transfer skills through an LL environment^44,45^.

Overall, the analysis of the distribution of projects funded under the different Horizon schemes reveals several noteworthy insights into the role of transdisciplinarity in addressing major sustainability challenges and fostering transformative change. In particular, there is a marked discrepancy between the number of projects funded under the RIA scheme, which represents nearly half of the the sample, and those supported through the IA scheme. This finding suggests that, within the EC calls, transdisciplinarity - and, more broadly, the contribution of LLs - play a crucial role in needs assessment, knowledge exchange, and multi-actor co-creation during solution development, rather than in later stages where co-created innovations are translated into outputs more directly oriented towards market uptake. These discrepancies may reflect several barriers within LLs, foremost among them the difficulty of ensuring the scalability and replicability of the solutions developed. More generally, it is well established that the strong contextualisation of innovations constrains their scalability. Indeed, scalable solutions must address widely shared societal needs, whereas LLs typically operate not only as spaces for analysing specific end-user needs^20^, but also within particular ecosystems and under defined political agendas^21^. Similarly, to ensure the scalability and replicability of the solutions and knowledge generated, LLs should be grounded in robust intentional entrepreneurial design and implementation from the earliest phases of the project. This also includes prioritising clearly defined specific dissemination activities: achieving this requires LLs to draw on a range of expertise capable of supporting the technological maturation of the solutions developed, including entrepreneurial competences, skills in communicating project results, and the ability to provide external actors with training and capacity-building opportunities^21^, all of which are key priority activities and competences of the CSA scheme, which is not primarily oriented towards technological development^21^.

The differences among the funding schemes of the projects are also reflected in the terminology adopted (columns eleven and twelve of the database). Table 3 lists all the terms, classified into four broad macro-areas: *living lab typologies; hubs, networks, and multi-actor infrastructure; pilots, demonstrators and case studies; and platforms and innovation ecosystems*.

**Table 3:** List of terms associated with “Living Lab” reported in the description of the different H2020 and HEU-funded projects related to the agri-food system. Authors’ elaboration based on results deriving from the systematic project review.

LIVING LAB TYPOLOGIES								
Living Lab(s), Living Laboratory(-ies)		Participatory Living Laboratories, Co-creation Living Labs, Multi-objective and Multi-actor Living Labs		Living Lab-like Structure, Living Lab Approach		City Labs, Fab Labs, Farm Labs, Local Living Labs, City Living Labs, Rural Living Lab, Real-world Experimental Living Labs, Regional Living Lab		Policy Labs, Policy Innovation Labs, Research and Innovation Labs
Food System Labs, Agroforestry Living Labs, Agroecology Living Labs, Soil Health Living Labs, Nutrient-oriented Living Labs		Pan European Investors’ Living Labs, European Network of Agroecological Living Labs, Network of Agroecology Living Labs European Hub of Co-creating Local Food System Labs, Network of Living Labs, European Network of Living Labs and Research Infrastructure		Innovation Labs, Accelerator Labs, Enabler Labs, Replication Lab, Result-driven Labs, Living Lab-based Open Schooling, Socio-ecological Women Innovator Living Labs, Living Lab Value Chains		Iot/5 g Lab, Lighthouse(s), Dynamos, Stakeholder Groups, AKIS Actors, AKIS, AKIS Ecosystem, AKIS Network, AKIS Stakeholders, Scar-AKIS, National Horse AKIS, Micro-AKIS, AKIS Governance		Multi-actor Agroecology Transdisciplinary Living Labs, Multi-stakeholder Living Lab Innovation Production Systems
**HUB, NETWORKS AND MULTI-ACTOR INFRASTRUCTURE**								
Hubs		Innovative Practice Hubs, Excellence Hub, Capacity Building Hub		Multi-actor Farmer Cluster, Multi-actor Team		Thematic Network(s), Innovation Networks, Cooperation and Innovation Networks, Multi-actor Thematic Network, Multi-actor Food System Interactive Thematic Networks		
Innovation Hubs, Communication and Knowledge Hubs, Transformation Hub, Innovation and Collaboration Hubs, Digital Innovation Hubs, Virtual Innovation Hubs, Biomass Innovation Design Hubs, Food Hubs, Agridemo-hub		Multi-actor Network, Demo-farm Network, Forest and Agriculture Networks, Pilot Network		African Hub Cities, Regional Hubs, Regional Cluster (Hubs), National Innovation Hubs, Local Innovation Hub, National Bioeconomy Hubs, Rural-urban Learning Hub, City-region Food System Hubs		National Level Innovation Networks, Multi-actor Eu-wide Thematic Network, Regional Agroforestry Innovation Networks, European Agroforestry Association National Network, European Rural Bioeconomy Network, European Interregional Network, Multi-actor Interactive and Innovation-driven Expanded Agroforestry Network, Forest And Agriculture Networks, Pan-European Multi-actor Network, Pan-European Network of Food System Science, Pan-African Network for Economic Analysis Of Policies, Common Agricultural Policy Network, Transnational Ecosystem		
Multi-actors, Multi-actor, Multi-stakeholder Participatory Approach, Multi-actor Innovation Hubs, Multi-actor Innovation Interregional Transversal Hubs, Multi-actor Approach, End-user Multi-actor Approach, Multi-actor and Participative Approach, Multi-actor Co-creation Approach, Multi-actor Systemic Approach, Multi-actor Governance, Multi-actor Collaboration						Cooperation Networks, Open and Participatory Network of Cities		
**PILOTS, DEMONSTRATORS, AND CASE STUDIES**								
Pilot, Pilots, Pilot Cases, Pilot Sites, Pilot Plots, Real World Pilots, Pilot Initiatives, Innovative Pilot Experiences, Pilot Areas, Co-designed Pilots	Case Studies, Field Case Study, Golden Cases, Demonstration Case Studies, Co-innovation Case Study, Pioneering Case Studies		Multi-actor Case Studies, Participatory Case Studies, Co-innovation Case Study, Case Study Hubs, Thematic Case Study Hubs			Demo Cases, Demo Sites, Demonstration Farms, Demo Farms, Pilots as Lighthouse Demonstrators, Lighthouse Demo’s, Lighthouse Demonstrators, Lighthouses Projects		
Use Cases, Test-bed(s), Study Areas, Demonstration Plots			Pilot Regions, Cross Regional Pilot			Sustainable Innovation Pilots		
**PLATFORMS AND INNOVATION ECOSYSTEMS**								
Transdisciplinary Approach, Multi-actor Approach(es), Multi-actor Framework, 3-tier Multi-actor Approach, Multi-actor and Multidisciplinary Approach, Multi-actor Transdisciplinary Approach, Multi-actor Structures, Multi-actors, Multi-actor Consortium, Multi-actor Innovation Partnership Networks, Multi-actor Innovation Partnership Groups, Pilot Multi-actor Territorial Networks, Operational Groups, Eip Agri Operational Groups, Regional Multi-actor Operational Group, Regional Multi-actor Partnerships, Multi-stakeholder Network(s), Multi-stakeholder Approach, Multi-stakeholder and Interdisciplinary Approach							Stakeholder Advisory Board, Participatory Nexus Dialogues, Multi-stakeholder Dialogues, Multi-level Stakeholder Dialogue, Multiple-stakeholder Dialogues	
Open Innovation Ecosystem, Multi-actor Rural Innovation Ecosystems, Solar Energy Innovation Ecosystem, Symbiotic Ecosystem, Urban Food Systems and Ecosystems					Innovation Platforms, Stakeholder Platforms of Trust, Rural Bioeconomy Platforms, Multi-actor Platforms, European Multi-actor Platform, Network of Farms and Experiential Platforms		Urban Agriculture Forum, Stakeholder Forums, Multi-actor Consultations	
Community(-ies) Of Practice					Innovation Platforms, Stakeholder Platforms of Trust, Rural Bioeconomy Platforms, Multi-actor Platforms, European Multi-actor Platform, Network of Farms and Experiential Platforms			

Elaboration based on qualitative data extracted from the project abstracts collected within the CORDIS repository. Terms are grouped under four main areas and are separated by a comma.

From a terminological perspective, we observe that some projects do not adopt any LL-related terminology, whereas others employ terms that recur across projects funded under different schemes (e.g., “living lab,” “hub,” and “lighthouse”). However, our experience shows that, in some cases, terminology can vary according to the type of funding scheme. For example, in RIAs, terms such as “case study” and “community of practice” are common, as are “dialogue” and “stakeholder forum.” In IAs, terminology more frequently refers to mechanisms of acceleration and replicability, including “demonstration,” “accelerator/enabler,” and “exploitation panel” or “pilot”. At the same time, in projects funded under the CSA scheme, there is extensive use of terms related to dissemination and knowledge exchange, such as “network,” “community of practice,” “platform,” and “AKIS.”

## Technical Validation

The results emerging from our study underwent multiple stages of validation, as detailed in the methodological section. In particular, although CORDIS project abstracts represent a valuable entry point for exploring the extent to which LL approaches are reflected across projects, our experience indicates that consulting secondary sources is necessary to ensure a more reliable selection of projects. Indeed, the exclusive use of project abstracts available in the CORDIS repository may not enable a rigorous distinction between the implementation of an LL and that of a more generic multi-actor approach within projects.

The robustness and validity of this database are ensured first and foremost by the scientific rigour underpinning the selection and validation steps carried out as part of our systematic project review. Moreover, we implemented additional measures to verify the results after the review process. In particular, we triangulated information on the selected H2020 and HEU projects with reports, strategies, and other documents produced by the EC. Among the initiatives reviewed, there are:

- The “Food2030 Project Family” cluster, which brings together and promotes collaboration among projects operating in urban contexts, including Food2030, FoodShift 2030, FoodE, Food TRAILS, Cities 2030, and FUSILLI, in order to ensure the long-term sustainability of the local LLs activated by each of the projects^47^.
- “The European Partnership Accelerating Farming Systems Transition: Agroecology Living Labs and Research Infrastructures”, which includes several H2020 projects, such as ALL-Ready, AE4EU, and SMS, and seeks to consolidate LLs and other initiatives launched under the Mission Soil of the HEU, beginning with the PREPSOIL and FOODPATHS projects^48^.

Most of the projects identified in this study were funded under the H2020 framework programme. It was within this funding cycle that several project clusters, such as those noted above, emerged, providing both the theoretical underpinnings and the first practical applications that informed and enabled follow-on initiatives funded under HEU. This trajectory suggests a degree of programmatic and conceptual continuity in EC funding.

We are aware that the database generated from this research will require continuous refinement and periodic updating. In this regard, the terminology extracted from the various project abstracts has supported the development of a precise search string with the aim of facilitating the identification of relevant projects applying the LL approach within the CORDIS repository. With appropriate temporal adjustments, our search string, together with the methodology provided, could facilitate the retrieval of projects funded after the period examined in our study. The search string to be used is as follows: contenttype = ‘project’ AND framework programme = ‘HORIZON’,‘H2020’ AND language = ‘en’ AND ((‘AGRI*’ OR ‘AGRO*’ OR ‘FOOD*’) AND (‘LAB*’ OR ‘HUB*’ OR ‘MULTI-ACTOR*’ OR ‘MULTI-STAKEHOLDER*’ OR ‘ECOSYSTEM*’ OR ‘DYNAMO*’ OR ‘PANEL*’ OR ‘PILOT*’ OR ‘DEMO*’ OR ‘LIGHTHOUSE*’ OR ‘TEST-BED*’ OR ‘FORUM*’ OR ‘NETWORK*’ OR ‘PLATFORM*’ OR ‘AKIS*’ OR ‘GROUP*’ OR ‘PARTNERSHIP*’ OR ‘ALLIANCE*’ OR ‘CLUSTER*’ OR ‘INTERFACE*’ OR ‘ADVISORY BOARD*’ OR ‘CONSULTATION*’ OR ‘DIALOGUE*’ OR ‘COMMUNITY OF PRACTICE*’ OR ‘CASE STUD*’ OR ‘STUDY AREA*’ OR ‘USE CASE*’)).

## Usage Notes

By mapping and analysing the main characteristics of EU-funded projects, this database offers a body of practical experiences that is particularly valuable at a time when place-based research is needed on the potential and limitation of LLs for the sustainability of our agri-food systems^24,25^. First and foremost, the database is intended to serve as a resource not only for European stakeholders, but also for audiences beyond Europe. Indeed, many of the EC-funded projects selected involve cross-continental partnerships, especially with African partners and communities. In general, the column reporting the societal issue addressed by each project may help identify projects that address specific issuess potentially experienced in other contexts and inform the pathways towards possibile solutions.

Moreover, this database could be used to compare the characteristics of LLs across the funding programmes analysed in this study with those observed in other programmes, including additional EC funding programmes (e.g., Erasmus+), and potentially beyond. To this end, the search string developed by this study could be applied to retrieve projects funded under other programmes. Comparative analyses could then be conducted by using the database columns that capture the characteristics of the LLs implemented across projects.

One of the main findings of this study concerns the uneven distribution of funded projects across the various funding schemes. In the context of agri-food systems, the LL approach appears to be promoted mainly under the research-oriented RIA scheme, while it is far less prevalent in the more market-oriented IA scheme.

This finding calls for policy considerations on the possible extension of LL approaches within IA funding scheme and, more broadly. on the role of transdisciplinarity in supporting sustainable transitions. The EC's emphasis on LL-oriented activities predominantly within RIAs suggests a strategic focus on transdisciplinarity during the co-design and early innovation development phases. As a result, the technological maturity of the proposed solutions often appears to remain at an exploratory stage, rather than advancing towards replication and scalability. This raises important questions regarding the role and effectiveness of LLs aimed at market uptake, and points to a relevant direction for future research: *within IA-funded projects, designed to bring innovation closer to the market, what outcomes and impacts do LLs actually produce?*

A comparative analysis of the LLs implemented across multiple projects can provide a robust empirical basis for identifying the critical factors associated with open innovation. This is particularly relevant,, although not exclusively, for projects funded under schemes oriented towards market deployment, as it may generate both managerial and operational insights into how innovations are designed,accepted, used, and adapted by society. Within the HEU framework, the concept of “societal readiness” offers a reference point for Responsible Research and Innovation, as it highlights the extent to which - research outputs are aligned with articulated societal needs and, consequently, their potential for uptake and sustained use^49,50^. At the same time, such alignment constitutes a foundational principle of the LL paradigm itself, which is explicitly based on co-creation with societal users. For this reason, investigating the relationship between technology maturity, societal readiness, and their underlying determinants, is especially relevant in the context of LLs, and may contribute to a more evidence-based understanding of how innovation can effectively progress towards societal uptake.

Another promising avenue for future research lies in examining the social and environmental needs that EC-funded projects have sought to address. Such an analysis could shed light on the challenges that are currently perceived as most urgent for driving a transition towards more sustainable practices. Within the broader post-growth perspective applied to agri-food systems, we consider the use of *Doughnut Economics*^4,51^ to be particularly relevant. This framework approaches well-being from a holistic perspective, by systemically situating societal well-being (articulated through the 12 essential needs -water, food, health, education, income and work, peace and justice, political voice, social equity, gender equality, housing, networks, and energy) within planetary boundaries(captured through the 9 ecological ceilings:climate change, ocean acidification, chemical pollution, nitrogen and phosphorus loading, freshwater withdrawals, land conversion, biodiversity loss, air pollution, and ozone layer depletion). Given that this framework has been widely applied through multi-actor approaches, and across multiple domains like sustainable urban planning, (as in the case of Amsterdam^51^, or in the evaluation and improvevent oftourism stretgies^52^, we believe it could also be effectively applied at the level of individual projects within our database. A similar application could support not only a better classification of the type of the challenges tackled by the different projects, but also enable further analyses of the relationship between the specific challenges and the characteristics assumed by LLs across projects.

## Data Availability

Our dataset is available for download, in its current second version, as an open access Excel file within the Zenodo repository at the following link: 10.5281/zenodo.18538508^43^. The dataset is licensed under a Creative Commons Attribution 4.0 International License, which permits use, sharing, adaptation, distribution, and reproduction in any medium or format, provided that appropriate credit is given to the original authors and the source of the information retrieved, a link to the license is provided, and any changes made are indicated. The full terms of the license are available at: http://creativecommons.org/licenses/by/4.0/.

## Code availability

No code has been applied to this work.
